# Intraoperative portal vein insulin assay combined with occlusion of the pancreas for complex pancreatogenous hypoglycemia: Two cases report

**DOI:** 10.1097/MD.0000000000003928

**Published:** 2016-07-01

**Authors:** Zhiying Yang, Haidong Tan, Yongliang Sun, Shuang Si, Li Xu, Xiaolei Liu, Liguo Liu, Wenying Zhou, Jia Huang

**Affiliations:** Department of Hepatobiliary Surgery, China-Japan Friendship Hospital, Beijing, China.

**Keywords:** intraoperative portal vein insulin assay, multiple endocrine neoplasia, nesidioblastosis, pancreatogenous hypoglycemia, surgical strategy

## Abstract

Intraoperative localization and confirmation of complete resection of the hypersecreting tissue are the 2 main challenges in the management of pancreatogenous hypoglycemia. Here, we report our experience with intraoperative portal vein insulin assay combined with occlusion of the pancreas in the management of pancreatogenous hypoglycemia. Clinical courses of 2 patients with biochemical evidence of a pancreatogenous hypoglycemia were studied. The preoperative diagnosis was multiple endocrine neoplasia 1 (MEN-1) and nesidioblastosis, respectively. Rapid intraoperative portal vein insulin assay combined with occlusion of the pancreas was used to localize and confirm complete excision of the hypersecreting tissue. Hypoglycemia was successfully treated in both the patients. In the MEN-1 patient, 2 small tumors in the head of pancreas were not resected, as they were deemed noninsulin secreting by intraoperative portal vein insulin assay, thus avoiding a total pancreatectomy. In the patient with nesidioblastosis, using intraoperative portal vein insulin assay combined with occlusion of the pancreas, an appropriate amount of pancreatic tissue was resected thereby avoiding recurrence and diabetes. This technique may be of particular value in patients with complex conditions such as MEN-1 and nesidioblastosis, to localize and achieve complete resection of hypersecreting pancreatic tissue.

## 1 Introduction

Pancreatogenous hypoglycemic syndrome is a disorder that occurs as a consequence of inappropriate and unregulated secretion of insulin by pancreatic β-cell tumors (insulinomas) or by nesidiodysplastic β-cells presenting as noninsulinoma pancreatogenous hypoglycemic syndrome. It may also be associated with multiple endocrine neoplasia (MEN) syndromes. Preoperative localization of abnormal insulin secreting tissue remains challenging due to its small size and often the diffuse nature of the disease in the pancreas. Conventional cross sectional imaging and endoscopic ultrasound (EUS) though helpful, often fail to localize pancreatic abnormalities in noninsulinoma pancreatogenous hypoglycemia. Selective intra-arterial calcium stimulation with hepatic venous sampling is useful in the preoperative mapping of the disease process within the pancreas; however, this is not helpful in determining the completeness of surgical resection. Intraoperative blood glucose monitoring is routinely used to determine the completeness of excision of involved tissue,^[1,2]^ but this is very prone to misinterpretation in patients with multiple lesions and noninsulinoma pancreatogenous hypoglycemia. There are only a few reports of intraoperative rapid insulin assay for this condition in the literature.^[3–6]^ Here, we present 2 cases of MEN-1 and nesidioblastosis respectively, with symptoms of hypoglycemia. We successfully carried out intraoperative portal venous insulin assay combined with occlusion of the pancreas to localize the pathology and determine the adequacy of resection in these patients.

## 2 Materials and methods

### 2.1 Operative procedure

A standardized surgical exploration was performed in both cases. Exhaustive visual inspection and palpation of the whole peripancreatic area, including the liver was carried out. After full Kocherization, the portal vein was exposed and the posterior aspect of the body/tail of the pancreas was also dissected. The whole pancreatic gland was manually palpated and further examined with enhanced intraoperative ultrasound (IOUS) to determine the presence and location of pancreatic lesions. If a lesion was found, 1 mL blood samples were drawn simultaneously from the portal vein and peripheral veins 20 minutes before and after tumor enucleation. If no tumor was found, the splenic artery and vein were dissected at the level of the body of pancreas. One milliliter blood samples were drawn simultaneously from the portal vein and peripheral vein before and after 1 minute of clamping the pancreas/splenic vessels, respectively. Rapid insulin assay was performed in the samples and further intraoperative surgical strategy was determined based on the results.

### 2.2 Intraoperative rapid insulin assay

Peripheral blood glucose levels were measured by a blood glucose meter (ONETOUCH Ultravue, Shanghai, China). Peripheral and portal vein blood insulin levels were measured using the Sandwich method by a rapid intraoperative assay (Roche Diagnostics GmbH, Mannheim, Germany). Initially, 20 μL human serum, antiinsulin monoclonal antibody, and ruthenium labeled antiinsulin monoclonal antibody were mixed together to get sandwich composites. Then particles coated with streptavidin were placed in the intermixture in order to combine with the sandwich composites through the reaction between biotin and streptavidin. The whole intermixture is then placed into a measuring cell. The particles are adsorbed onto the electrodes by a magnet. The noncombined materials are washed away by Procell/Procell M. The chemiluminescence produced by the electrode pressure is read by a photomultiplier. The final results are obtained from the standard curve by the machine automatically. The whole procedure takes around 18 minutes.

## 3 Case presentation

### 3.1 Case 1

**Figure 1:**
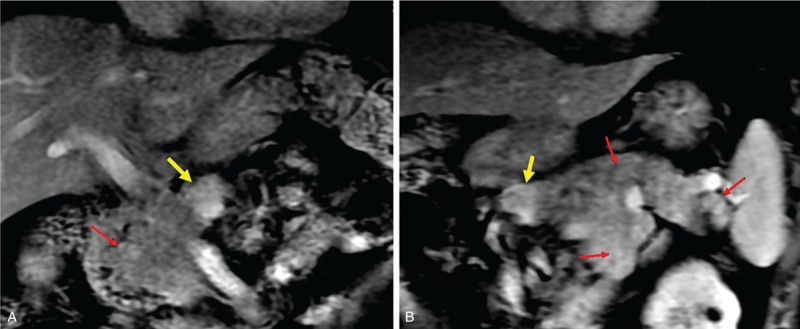
The preoperative magnetic resonance imaging of Case 1 showed multiple tumors in the whole pancreas gland. Yellow arrows: the tumor localized in neck of pancreas; red arrows: the multitumors localized in pancreas.

**Figure 2:**
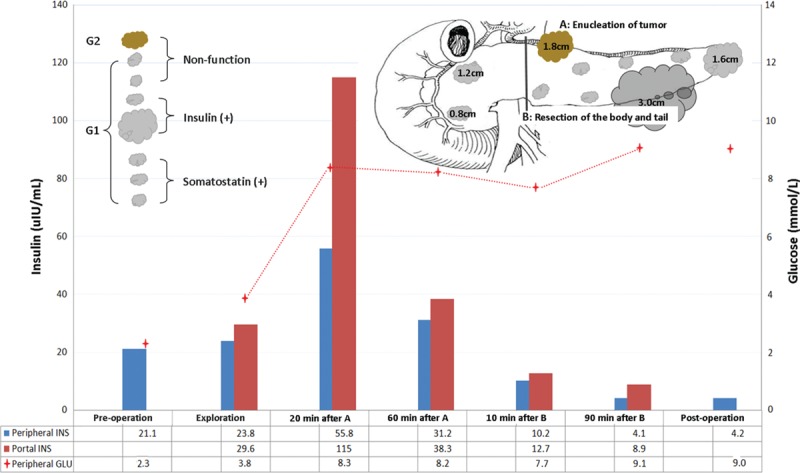
The intraoperative insulin assay of peripheral and portal vein and operative decisions for Case 1. INS = insulin, GLU = blood glucose.

A 36-year-old male presented with a 3-month history of symptoms of hypoglycemia (Whipple triad). He had history of MEN-1 with verrucous nodules on the skin, pituitary tumor, parathyroidectomy, and subtotal thyroidectomy in the past. Although serum calcium normalized following his neck surgery, he started to present with intermittent symptoms of hypoglycemia such as seizures associated with blood glucose levels of 1.6 to 1.8 mmol/L and serum insulin levels of 18.6 to 27.6 uIU/mL (I/G ratio: 0.63–0.85). The imaging examination (ultrasound, computed tomography and magnetic resonance imaging) all showed multiple tumors in the pancreas (Fig. 1). A laparotomy was performed in March 2015. The intraoperative rapid insulin assay and operative decisions are presented in Fig. 2. During the exploration, multiple tumors were confirmed within the whole pancreas gland. The tumor in the neck of pancreas was harder than others and was initially enucleated. After excision of this tumor from the neck of pancreas, blood glucose normalized, however, the blood insulin levels in the peripheral and portal venous blood did not decrease, but steadily continued to increase. Hence the body and tail of pancreas was excised. Pathology confirmed 7 pancreatic endocrine nodules, of which 2 were positive for insulin, 3 positive for somatostatin, and 2 negative nodules. Two other nodules were found in the head of pancreas on IOUS (0.8 and 1.2 cm, respectively). However, after removal of the body and tail of the pancreas, blood glucose, peripheral blood insulin, and portal venous blood insulin returned to normal levels. Thus it was felt that these 2 nodules in the head of the pancreas were noninsulin-secreting tumors and hence a total pancreatectomy with its associated morbidities was not considered. The patient recovered uneventfully and was discharged 11 days postoperatively. At follow-up for 6 months later, the fasting glucose levels were 7.2 to 8.3 mmol/L.

### 3.2 Case 2

**Figure 3:**
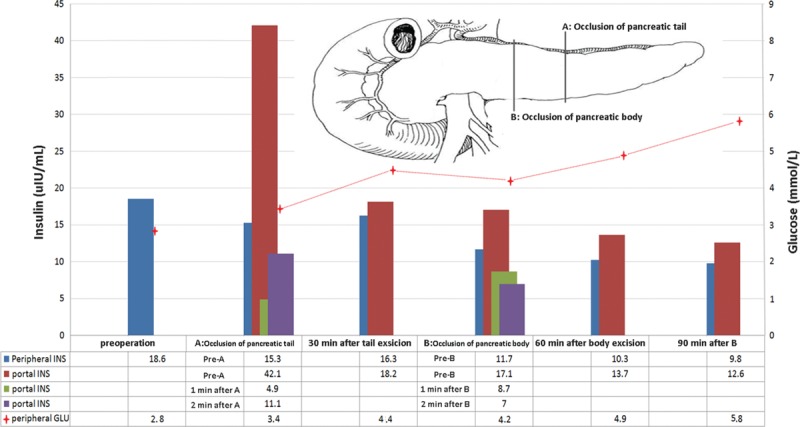
The intraoperative insulin assay of peripheral and portal vein and operative decisions for Case 2. INS = insulin, GLU = blood glucose.

A 29-year-old female presented with symptoms of intermittent hypoglycemia of 3 years duration. During this time she had put 15 kg in weight. The blood glucose level during seizure episode was 1.9 mmol/L and postprandial blood glucose measured at 3 mmol/L. The fasting glucose to insulin ratio was more than 0.3 during all her admissions. The imaging examinations did not show any pancreatic lesions but just uniform enlargement of the pancreatic body and tail. Presence of microinsulinomas or nesidioblastosis was suspected. Laparotomy was performed in May 2014. There was no any tumor found on palpation and IOUS. The intraoperative rapid insulin assay results and operative decision are presented in Fig. 3. The portal vein insulin levels rapidly decreased after occlusion leading us to conclude that the hypersecreting tissue of pancreas was indeed in the body and tail. The patient recovered uneventfully following distal pancreatectomy and was discharged 9 days postoperatively. The histopathological examination confirmed nesidioblastosis. She remains asymptomatic with fasting blood glucose levels of 4.3 to 5.2 mmol/L at 16 months following surgery.

## 4 Discussion

Insulinoma is the most common cause of pancreatogenous hypoglycemia. It has an incidence of 1 to 4 per million per year, and most (80%–90%) cases are caused by solitary benign adenomas, nearly always exclusive to the pancreas. Approximately 5% of cases are associated with MEN-1.^[7]^ Nesidioblastosis, even though unusual in adults, is described as an occasional cause of pancreatogenous hypoglycemia.^[8,9]^ The ideal treatment of pancreatogenous hypoglycemia is complete surgical excision of the hypersecreting tissue.

With typical Whipple triad and a high serum insulin level, the diagnosis of pancreatogenous hypoglycemia is not difficult. However, it is more difficult to localize the small and often multiple tumors, or to confirm nesidioblastosis. Preoperative localization studies combined with IOUS and pancreatic palpation can successfully localize insulinoma in 89% to 97% of patients.^[10,11]^ However, 4% to 16% of tumors are not recognized intraoperatively.^[4,9,12]^ Another intraoperative challenge is the determination of completeness or adequacy of resection of the hypersecreting tissue. Intraoperative blood glucose monitoring after tumor excision is often used to determine operative outcome, but this technique has a variable accuracy ranging from 56% to 81%.^[1,2,4,11,12]^ Intraoperative blood glucose monitoring is a comprehensive end product that reflects the overall blood glucose regulatory mechanisms in the body. This result may sometimes be influenced by factors other than insulin such as stress response to surgery. Some MEN-1 patients may have multiple tumors and different tumors may secrete different hormones such as glucagon, somatostatin, etc. Thus the blood glucose levels may not accurately reflect the response to insulin. The false-negative rate may be as high as 23%.^[1]^ Meanwhile, the elevation of the blood glucose levels may commonly occur as long as 30 to 90 minutes after tumor excision. So because of these limitations, intraoperative blood glucose monitoring is not an ideal test for assessing completeness of resection. As a result, intraoperative measurement of insulin has been studied to assess completeness of resection.^[3–6,11]^

Besides the reports of Proye et al^[4]^ and Amikura et al,^[11]^ most studies used the peripheral blood for insulin monitoring. In Proye study, the blood insulin level of peripheral and portal vein decreased to the normal level in 75% of the patients. In Amikura study, the success rate for peripheral blood glucose monitoring was 59% (8/14), 70% (7/10) for peripheral blood insulin monitoring and100% (4/4) for portal vein insulin monitoring. Scott-Coombes study^[5]^ found that blood insulin monitoring was not ideal in this regard. In Carneiro study,^[6]^ the accuracy rate of intraoperative peripheral blood insulin monitor was 89%. The difference in the results of peripheral blood monitoring may be due to the first pass metabolism of insulin through the liver. Thus peripheral blood insulin levels are not reflective of portal venous insulin levels and might not accurately determine completeness of resection of hypersecreting pancreatic tissue.

In our first patient, MEN-1 and multiple pancreatic tumors were diagnosed preoperatively. During the exploration, multiple tumors were confirmed within the whole pancreas gland. The tumor in the neck of pancreas was harder than others and was initially enucleated. After excision of this tumor from the neck of pancreas, blood glucose normalized, however, the blood insulin levels in the peripheral and portal venous blood did not decrease, but steadily continued to increase. This result illustrates the limited usefulness of blood glucose monitoring. The blood insulin results suggested the presence of functional insulinomata and hence the body and tail of pancreas was excised. Pathology confirmed 7 pancreatic endocrine nodules, of which 2 were positive for insulin, 3 positive for somatostatin, and 2 negative nodules. Two other nodules were found in the head of pancreas on IOUS (0.8 and 1.2 cm, respectively). However, after removal of the body and tail of the pancreas, blood glucose, peripheral blood insulin, and portal venous blood insulin returned to normal levels. Thus it was felt that these 2 nodules in the head of the pancreas were noninsulin-secreting tumors and hence a total pancreatectomy with its associated morbidities was not considered. This decision would not have been possible without peripheral and portal venous rapid insulin assays. Intraoperative diagnosis and localization in adult nesidioblastosis is very difficult. An empirical resection of the body and tail of the pancreas used to be performed by many surgeons when tumor could not be found on intraoperative palpation and IOUS. Alternative strategies using selective arterial calcium injection^[13]^ can roughly localization. But it was complicated in technique and has the potential for either incomplete resection or induce diabetes mellitus secondary to excessive resection. As illustrated in our paper, the portal vein insulin levels will respond rapidly to the occlusion of the body of pancreas combined with the splenic artery and vein. However, the peripheral blood glucose and insulin will respond only 15 to 30 minutes later. This method has the potential to rapidly and correctly localize the position of hyper secreting tissue in the pancreas gland. In our second case, the portal vein insulin levels rapidly decreased after occlusion leading us to conclude that the hypersecreting tissue of pancreas was indeed in the body and tail. This patient recovered without any complications with a normal fasting blood glucose (4–5 mmol/L). In Proye report, 1 nesidioblastosis patient recurred 3 months postoperatively, even though the intraoperative blood glucose returned to normal levels, but with high portal vein insulin levels. Thus our method will not only help to localize the resection, but also determine the extent of resection to avoid the incomplete or excess resection.

The 2 cases demonstrate that rapid intraoperative portal vein insulin assay with or without pancreatic/splenic vessel occlusion may be helpful to make the right intraoperative surgical decision. This method may be of particular value in patients with complicated conditions such as MEN-1 or nesidioblastosis.

## Acknowledgments

The authors would like to thank Dr. Raaj Kumar Praseedom in University Department of Surgery of Addenbrooke's Hospital for his assistance in the preparation of this manuscript.
